# Searching for a Toxic Key to Unlock the Mystery of Anemonefish and Anemone Symbiosis

**DOI:** 10.1371/journal.pone.0098449

**Published:** 2014-05-30

**Authors:** Anita M. Nedosyko, Jeanne E. Young, John W. Edwards, Karen Burke da Silva

**Affiliations:** Flinders University of South Australia, Faculty of Sciences and Engineering, Adelaide, South Australia, Australia; Aristotle University of Thessaloniki, Greece

## Abstract

Twenty-six species of anemonefish of the genera *Amphiprion* and monospecific *Premnas,* use only 10 species of anemones as hosts in the wild (Families: Actiniidae, Stichodactylidae and Thalassianthidae). Of these 10 anemone species some are used by multiple species of anemonefish while others have only a single anemonefish symbiont. Past studies have explored the different patterns of usage between anemonefish species and anemone species; however the evolution of this relationship remains unknown and has been little studied over the past decade. Here we reopen the case, comparing the toxicity of crude venoms obtained from anemones that host anemonefish as a way to investigate why some anemone species are used as a host more than others. Specifically, for each anemone species we investigated acute toxicity using *Artemia francisca* (LC_50_), haemolytic toxicity using ovine erythrocytes (EC_50_) and neurotoxicity using shore crabs (*Ozius truncatus)*. We found that haemolytic and neurotoxic activity varied among host anemone species. Generally anemone species that displayed greater haemolytic activity also displayed high neurotoxic activity and tend to be more toxic on average as indicated by acute lethality analysis. An overall venom toxicity ranking for each anemone species was compared with the number of anemonefish species that are known to associate with each anemone species in the wild. Interestingly, anemones with intermediate toxicity had the highest number of anemonefish associates, whereas anemones with either very low or very high toxicity had the fewest anemonefish associates. These data demonstrate that variation in toxicity among host anemone species may be important in the establishment and maintenance of anemonefish anemone symbiosis.

## Introduction

Marine animals are amongst the most venomous species on earth and the Cnidaria in particular are well known for the potency of their stings. Sea anemones are sessile organisms that possess a variety of proteic substances (such as peptides, proteins, enzymes, and proteinase inhibitors, see [1]) for protection, for hunting, and competitive interactions. Toxins are found in stinging cells called nematocysts, which fire semi-autonomously and have a sophisticated ability to recognize foreign animals [2]. Sea anemone venoms are complex polypeptides that cause a variety of toxic effects including lethality, haemolysis, and neurotoxicity [3]–[4] yet some species of fish and crustacean are able to tolerate anemone venoms and associate with anemones in a mutualistic relationship. The mechanisms involved in protecting anemonefish from anemone venom has been examined by multiple authors (e.g. [5]–[13]) since its discovery by Caspers in 1939. How anemonefish acquire immunity from stinging tentacles remains uncertain, however, it is generally agreed that the fish’s mucus plays an important role in its protection either through a blocking mechanism or through inhibition by mimicry (see [14] for a recent review).

Twenty-six species of anemonefish in the genus *Amphiprion* and monospecific *Premnas,* are found in only 10 species of anemones which act as hosts (Families: Actiniidae, Stichodactylidae and Thalassianthidae) [15]. Patterns of host anemone usage (in [15]; pg. 149, Table 1) indicate that some anemone species are preferred by anemonefish as they will compete for them [16]–[19], and they have large numbers of fish species associated with them (eg. *Entacmaea quadricolor*), whereas other anemones (*Heteractis malu* and *Cryptodendrum. adheasivum*) have only a single anemonefish species in association and will only be used if no other anemone is available. Hypotheses explaining the different patterns of relationship between anemonefish species and anemone species have been proposed by Fautin [16]–[17] and Murata *et al.*, [20] and include, olfaction (by fish), innate preference (by fish), competitive exclusion (between fish), and environmental requirements of the symbionts (both fish and anemone). Anemone use by different anemonefish species cannot be fully explained by innate or conditioned preference hypothesis [12], [15], [21], as some anemonefish are known to move from one anemone species as juvenile to a different anemone species as adults [22]–[24]. The claim that different anemone species must provide different fitness levels to fish however has been made, but what particular anemone attributes contribute to fish fitness has yet to be determined. Understanding the relationship between fish and anemone hosts and in some cases the extreme forms of specialization and generalisation by anemonefish has resulted in studies using multifaceted approaches such as phylogenetic analysis [25] and mathematical models [21] to decipher the complex pattern exhibited by this family of fish. These hypotheses have primarily focused on the relationship from the perspective of the fish whereas the influence of the anemone on this association has received less attention and may be key to understanding important aspects of the symbiosis.

**Table 1:** Morphological characteristics (Fautin and Allen, 1997) and number of anemonefish associated (Ollerton *et al* 2007) with each species of host anemone.

Species	Tentacle length (mm)	Oral Disc Diameter (mm)	# Anemonefish associates
*Entacmaea quadricolor*	100	50	13
*Heteractis aurora*	50	250	7
*H. magnifica*	100	500	14
*H. crispa*	75	1000	12
*H. malu*	40	200	1
*Macrodactyla doreensis*	175	500	4
*Stichlodactyla gigantea*	10	500	8
*S. haddoni*	10	500	7
*S. mertensi*	20	1000	13
*Cryptodendrum adhaesivum*	5	300	1

This rather complex symbiotic relationship requires an understanding of the requirements of both host (anemone) and symbiont (anemonefish) in order to understand the establishment and maintenance of the relationship [14]. Protection from predatory reef fish is the primary fitness benefit anemones gain by acting as a host to anemonefish, as proposed by Allen [26] and Fautin [18]. Anemonefish gain protection by living within the stinging tentacles of anemones [12], and this mechanism as outlined above remains unsolved. The physiological costs involved in protection must exist, however, the advantages gained for the fish are a long lifespan (anemonefish live up to 35 years whereas similar sized fish may only live between 5 to 10 [27]) and an increase in reproductive fitness (eg. egg protection [28]). To maximize fitness, anemonefish should choose anemone hosts that provide them with the highest quality refuge at the lowest cost to themselves with respect to physiological expenditure. If anemone quality varies, competition should exist for highest quality hosts, and is indeed the case as reported by Fautin [16] and others [17], [19]. Clearly, the question of ‘What defines a high quality anemone in the eyes of an anemonefish?’ must be explored.

Anemone morphology does differ amongst the 10 host species, primarily in overall size and in tentacle length (Table 1). Another characteristic that has clear variation is toxicity, and we believe this could be a critical factor in determining the quality as a host for anemonefish and may be responsible for limiting the number of anemone species, which can form a symbiotic relationship with anemonefish. Senčič and Maček [4] summarized the properties of venoms from fifteen different anemone species, none of which acts as a host for anemonefish, and found a significant difference in their lethal potency. A more recent review of 32 anemones [1] included one species (*Heteractis magnifica*) that hosts anemonefish found that its venom was a group II peptide of relatively high potency. Understanding the relative potency of the toxins among the host anemones will provide key information necessary to determine how anemonefish develop tolerance to the venoms, which in turn will provide insight into the existence and maintenance of the symbiotic relationship and how this association may have evolved.

## Materials and Methods

### 2.1 Anemone Collection

Anemones (*Cryptodendrum adhaesivum; Stichodactyla haddoni; Stichodactyla giagantea; Entacmaea quadricolor; Heteractis magnifica; Heteractis aurora; Heteractis crispa; Heteractis malu;* and *Macrodactyla doreensis*) were collected either from the coral reef fringing Lizard Island, Queensland, Australia (14°40′08″S 145°27′34″E) with the authority of the Great Barrier Reef Marine Park Association (GRMPA permit number G09/29194.1.) or obtained through an aquarium livestock trader that collects only from Queensland reefs (no permit required for buying anemones from aquarium shop). Animal collection and care were carried out in accordance with the Primary Industries and Resources, SA.

Nine of the ten anemonefish host anemone species were included in the study, the tenth species *Stichodactyla mertensii* we were unable to attain. Anemones were kept in aquaria in the laboratory in seawater and fed weekly with small pieces of fish or prawn, but were fasted for one week prior to venom collection.

### 2.2 Collection and Preparation of Venom

Venom was obtained using the milking technique, described in Senčič and Maček [4]. Unlike other methods of collection, which require a whole animal homogenate (e.g. [3], [29]) this method was used to preserve the life of the animal. The crude venom was obtained by gently massaging the tentacles of individual specimens within a plastic bag. Each individual was milked for venom 1–3 times and these venom samples were pooled and frozen at −80°C. Previous research has demonstrated that repetitive milking does not decrease the toxicological quality of the venom [4]. Anemone health was monitored and after two hours post milking, the anemone would open up to full size with no bleaching observed during or after the venom collection period.

Crude venom samples from host species were lyophilized and stored at −80°C until required for assays. Samples were resuspended in milli-Q water and assayed for protein content using the Thermo Scientific Pierce BCA protein assay kit with ovine serum albumin (BSA) used as a standard. Absorbance was read at 562 nm using a FLUOstar Omega spectrophotometer (BMGlabtech). Estimates of total protein concentration for each species were obtained from the BSA standard curve using GraphPad Prism v. 5.02 for windows (GraphPad Software, San Diego, California, USA). Relative ‘venom concentrations’ were based upon the total protein concentration in the crude venom and were used to determine dilutions for each toxicological assay.

### 2.3 Toxicity Endpoints

Toxicity tests were selected to represent the different qualities of venom that have been reported. These include cytolytic activity (using the haemolysis of sheep erythrocytes), whole organism lethality (using *Artemia francisca*), and neurotoxic activity (using limb paralysis in shore crabs, of the species *Ozius truncatus*).

#### 2.3.1 Erythrocyte haemolysis

Haemolysis measurements provide a rapid, easily reproducible, and quantifiable method for comparison of the cytolytic properties of different anemone venoms. Haemolytic assays were performed using ovine erythrocytes across a wide range of concentrations. Defibrinated blood was obtained from the Institute of Medical and Veterinary Science (Gilles Plains, South Australia) and stored at 4°C until required but was used within 3 days. Blood was centrifuged at 1000 g for 10 mins at 4°C. The supernatant and buffy coat were removed by aspiration and the cells washed three times with phosphate buffered saline (PBS). Following the final centrifugation, the resultant erythrocyte pellet was resuspended in PBS to make a 1% erythrocyte solution for haemolysis experiments. This concentration allowed the supernatant fraction of a totally lysed (100%) suspension of erythrocytes to record 0.8–1.35 absorbance units at 540 nm using a µQuant spectrophotometer (Bio-tek instruments Inc.). Total haemolysis was achieved by freezing and thawing the erythrocyte suspension three times.

A series of crude venom dilutions (0.1 ml) were applied to the erythrocyte suspensions (0.9 ml) and incubated at 37°C for 40 mins. After centrifugation at 1000 g for 2 mins at 4°C, the absorbance of the supernatant was measured at 540 nm to detect the release of haemoglobin. For all experiments, a positive control was obtained by the addition of 1% Triton to the erythrocyte suspension (according to Bailey *et al*. [30]) and a negative control was obtained using PBS. All assays were performed in duplicate for each host species with three replicates measured per concentration. Activity was expressed as percentage of haemolysis produced at each toxin concentration.

#### 2.3.2 Artemia lethality assay

Brine shrimp, *A. francisca* was utilized as a crustacean model to investigate the ability of sea anemone toxin to produce crustacean lethality. *A. francisca* cysts were obtained from the *Artemia* Reference Center, Ghent University, Belgium (www.aquaculture.ugent.be) and were hatched and harvested in accordance with the guidelines described in Vanhaecke and Persoone [31]. The standardized short-term *Artemia nauplii* toxicity test (ARC-Test) in Vanhaecke and Persoone [31] was used to assess the toxicity of crude host anemone venom. Ten brine shrimp (instars II and III reproductive stage) were transferred to a Petri dish and exposed to 10 ml of diluted venom and left overnight at 25°C. A range of anemone venom dilutions for each species were assessed on *A. francisca* with data expressed as percentage lethality. Reference tests and control experiments were performed using potassium dichromate [31] and filtered artificial seawater (salinity 36 ppt) respectively in place of venom.

#### 2.3.3 Shore crab neurotoxicity

All collections of shore crabs were authorised by a ministerial exemption from Primary Industries and Resources, SA (reference number 42000322). The number of crabs obtained for this experiment was kept to the minimum necessary for a meaningful interpretation of the data (on average 10 crabs per anemone species). The neurotoxicity assay was performed on the shore crabs (*Ozius truncatus*) collected from Marino Rocks, South Australia, with average carapace length of 2.5 cm, as described in Stándker *et al.* [29]. Crude venom dilutions were prepared with 0.43 M NaCl solution and 10 µL crude venom/gram of crab [32] was injected into the crabs at the junction between the body and the 3^rd^ walking leg. Crabs were placed on their dorsal surface and their ability to right themselves was recorded immediately and 20 minutes post injection. Anemone venom was applied to crabs in triplicate until the dosage preventing the righting response after 20 min exposure to the venom was reached. A positive control was obtained by injecting a crab with bioallethrin at 380 µg/kg of crab and 0.43 M NaCl at 10 µL/gram of crab as a negative control. All 94 crabs used in this study were treated according to collection permit, none were returned to the wild. Data were expressed as the effective paralytic dose range (highest non-paralytic to lowest paralytic dose).

#### 2.3.4 Overall toxicity

An overall toxicity measure was determined by ranking the toxicities of each of the host species in the three toxicological assays (lowest host toxicity for each assay scored 1, higher toxicity scored ≤n). These numbers were then tallied to obtain overall toxicity.

#### 2.3.5 Statistical analysis

For the *Artemia* lethality and erythrocyte haemolysis experiments the best-fit sigmoid dose response curve was determined using GraphPad Prism v. 5.02 for windows (GraphPad Software, San Diego, California, USA). Dose response curves were required to have correlation coefficients (r^2^) exceeding 0.75 to be included in further analysis. Anemone LC_50_ and EC_50_ values (venom concentrations lethal to 50% of tested *Artemia* and producing 50% haemolysis in erythrocytes respectively) and their standard error were determined from the best-fit dose response curves. Dose response curves were compared between anemone species using two-way analysis of variance. The shore crab effective paralytic dose ranges were compared by eye. The overall toxicity ranking was then correlated with the anemonefish association patterns for each anemone host species, essentially which fish species and how many of each fish species were found in association with each anemone species. Anemonefish association data was collected from Fautin and Allen [15] and Ollerton *et al.,* [21]. Spearman’s one-tailed correlation tests were considered statistically significant at p<0.05. To determine whether there was an overall statistically significant trend in the toxicities of host species across the three toxicological assays (p<0.05), Kendall’s coefficient of rank concordance W analysis was calculated [33].

The relationship between number of fish associates and overall toxicity ranking was compared using a linear model with a second order polynomial function fit to the model (R version ^©^).

## Results

**Figure 1:**
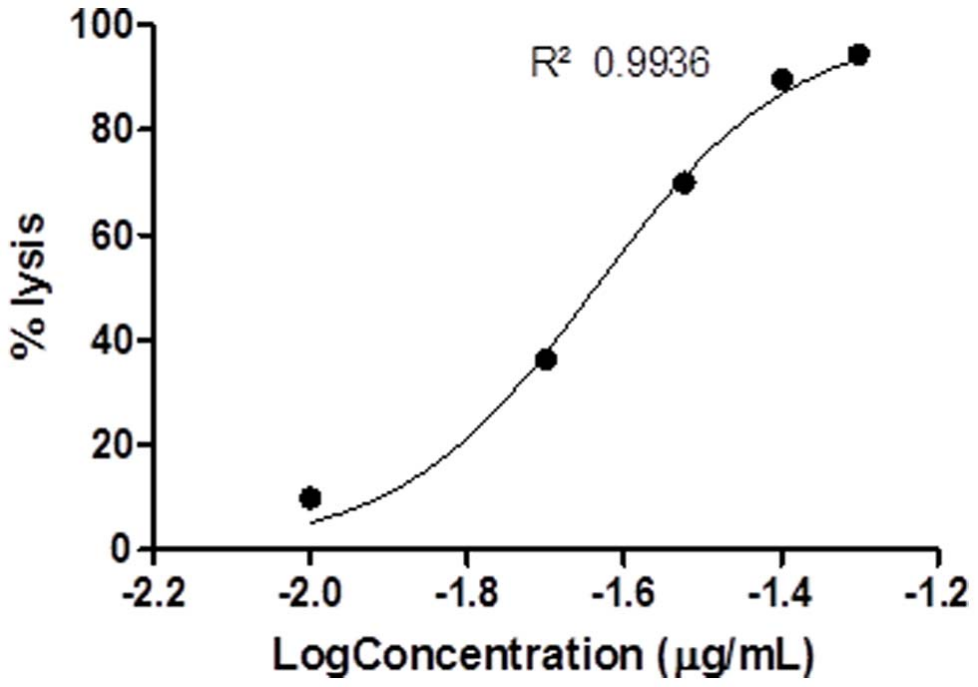
Haemolytic toxicity best-fit dose response curve for *S. haddoni*.

**Figure 2:**
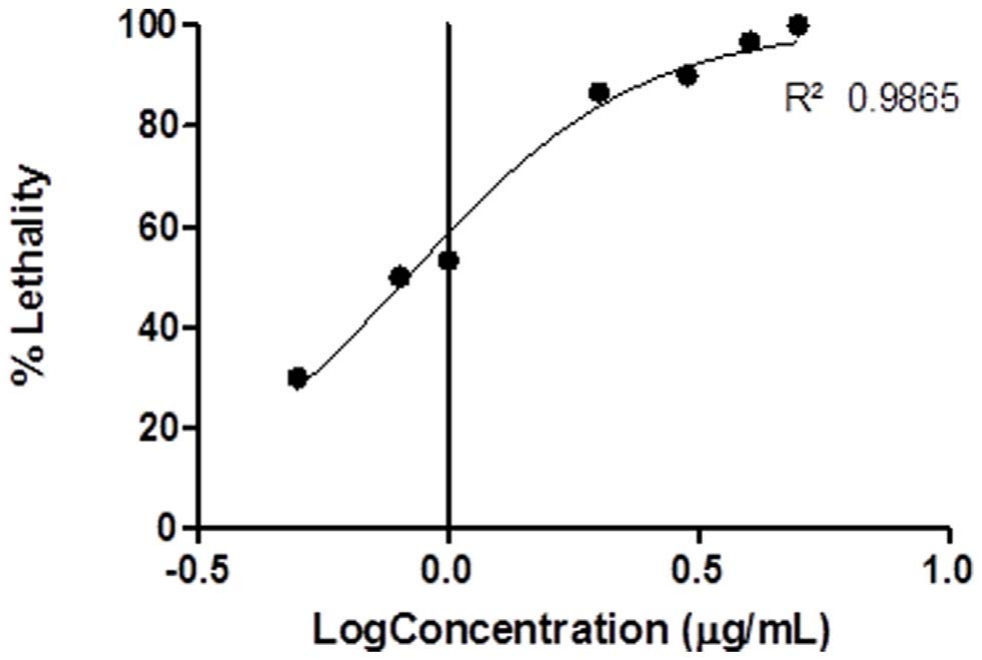
Acute toxicity (*Artemia* Lethality) best-fit dose response curve for *S. haddoni*.

**Figure 3:**
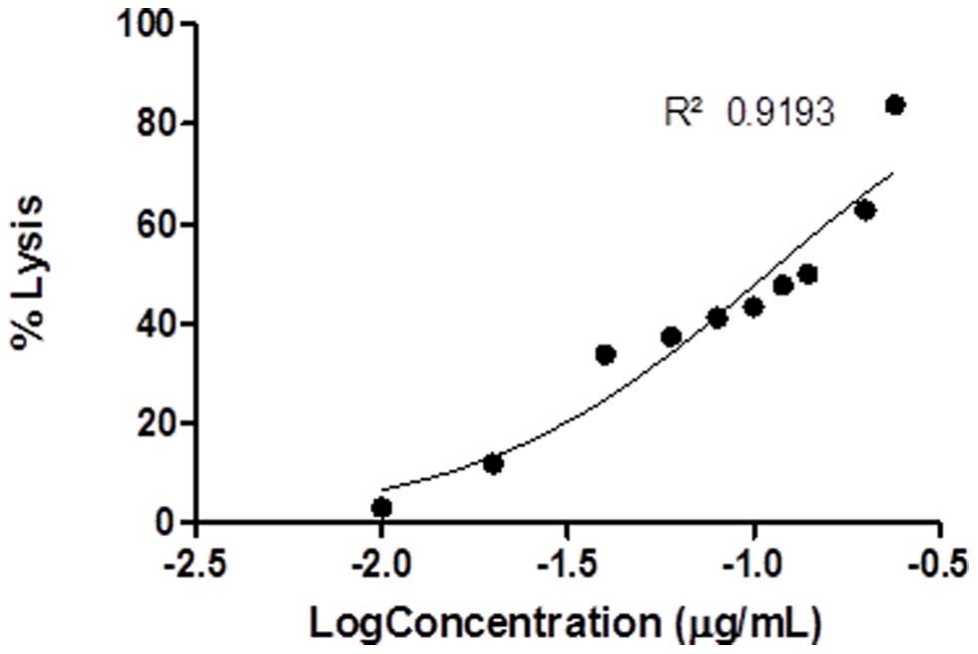
Haemolytic toxicity best-fit dose response curve for *H. magnifica*.

Nonlinear correlation coefficients (r^2^) of all dose response curves ranged from 0.76 to 0.99 (Tables 2 and 3) and these were all found to differ significantly from zero (t-test of fitted curve, p<0.05). In some cases (e.g. *S. haddoni*, for haemolysis, Fig. 1 and for *Artemia* lethality, Fig. 2) the dose response curves appeared monotonic, with curves closely representing the classical sigmoid shape. In other cases (e.g. *H. magnifica*, Fig. 3) the curve was more complex, with more than one turning point within the data, suggesting the venom may have contained more than one active toxin, or more than one mechanism of action [34]. In all cases, a single EC_50_ or LC_50_ was determined as an indicative measure of venom toxicity.

**Table 2:** Haemolytic best-fit dose response curve EC_50_ values (µg protein/mL) and the goodness of fit values r^2^ for anemone species.

Species	EC_50_	r^2^ value
*Heteractis malu*	>65	–
*Macrodactyla doreensis*	58.88	0.9323
*H. crispa*	6.47	0.9500
*H. aurora*	3.62	0.9520
*Entacmaea quadricolor*	0.62	0.8779
*Stichlodactyla gigantea*	0.32	0.9795
*Cryptodendrum adhaesivum*	0.14	0.9529
*H. magnifica*	0.11	0.9193
*S. haddoni*	0.02	0.9936

**Table 3:** *Artemia* lethality best-fit dose response curve LC_50_ values (µg protein/mL) for host species and the goodness of fit values only r^2^>0.75.

Species	LC_50_	r^2^ value
*Macrodactyla doreensis*	149.10	0.8160
*Heteractis malu*	1.84	0.9429
*H. crispa*	0.98	0.9827
*Stichylodactyla haddoni*	0.83	0.9865
*H. aurora*	0.51	0.9875
*Entacmaea quadricolor*	0.51	0.9229
*H. magnifica*	0.49	0.9079
*Stichylodactyla gigantea*	0.26	0.9757
*Cryptodendrum adhaesivum*	0.15	0.7661

### 3.1 Haemolysis

All host venoms exhibited haemolytic properties depending on the crude venom concentration applied to the ovine erythrocytes (Table 2; Fig. 1). A best-fit dose response curve comparison revealed significant differences (p<0.0001) in the estimated EC_50_ among all host species (Table 2).

Host venoms ranged significantly with the venom of *S. haddoni* found to be the most potent with 50% lysis occurring at 0.02 µg/mL and *H. malu* having the lowest haemolytic activity with 50% lysis occurring above 65 µg/mL, at which point undiluted venom extract was applied and an exact EC_50_ could not be obtained (Table 2).

### 3.2 *Artemia* Lethality Assay

All host species exhibited acute level toxicity on brine shrimp nauplii (*A. francisca*) (e.g. *S. haddoni*, Fig. 2). The venom of *C. adhaesivum* was found to be the most acutely toxic with 50% lethality occurring at 0.15 µg/mL and *M. doreensis* having the lowest acute toxicity with 50% lethality occurring at 149.1 µg/mL (Table 3), significantly lower compared to the other host species (p<0.0001). Significant differences in LC_50_ (p<0.0001) were also found among the other host species (Table 3).

### 3.3 Shore Crab Neurotoxicity

All host venoms tested exhibited neurotoxic activity in the shore crab, *O. truncatus* (Table 4). Neurotoxin effects ranged from slight twitching of leg at injection site to immediate severe and rigid paralysis. The severity of the paralysis observed after venom injection depended on the toxin concentration assayed with a definitive initial spastic and tetanic phase, and a later rigid phase observed in some individuals as described by Stándker *et al.* [29]. Although *H. malu* venom displayed no activity at the highest concentration given within the 20-minute experimental period (undiluted venom extract at 1500 µg protein/kg), within 24 hrs nerve paralysis was observed. This indicates that *H. malu* venom does contain a neurotoxin component, albeit with a lower toxicity. Four host species *C. adhaesivum, H. aurora, S. haddoni,* and *E. quadricolor* had equal effective dose ranges between 100 µg protein/kg to 150 µg protein/kg. Regardless of the neurotoxin effects within the 20-minute trial period, the majority of experimental individuals had developed severe paralysis within a period of 1–3 days, an indication of the considerable neurotoxic effect within the venom of host anemone species.

**Table 4:** Neurotoxin dose activity range (µg protein/kg) for host anemone crude venom in *O. truncatus*, at which point a crab has the ability to and does not have the ability to flip off its back after 20 mins post crude venom injection.

Species	Able to Flip	Not able to Flip
*Heteractis malu*	>1500	>1500
*Macrodactyla doreensis*	1300	1400
*H. magnifica*	1200	1300
*H. crispa*	700	800
*Stichlodactyla gigantea*	200	250
*Entacmaea quadricolor*	100	150
*H. aurora*	100	150
*S. haddoni*	100	150
*Cryptdendrum adhaesivum*	100	150

### 3.4 Overall Toxicity

A ranked analysis of the nine host anemones, based on the results of the three toxicity assays, indicates that *H. malu* venom has the lowest toxicity overall. In contrast, *C. adhaesivum* venom is most toxic in comparison to other host species and consistently exhibited either lower (*H. malu*) or higher (*C. adhaesivum*) activity across the three assays. Kendall’s coefficient of concordance *W* was 0.716 indicating that the ranked venom toxicities were generally concordant across all three assays (κ^2^ = 17.18, 8 degrees of freedom; p<0.05).

**Figure 4:**
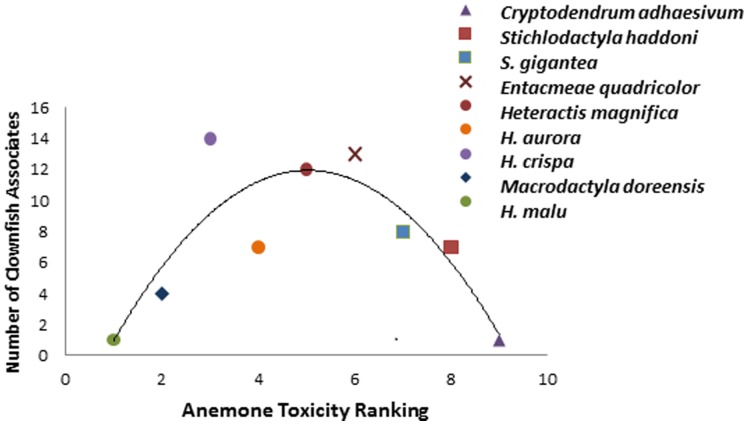
Relationship between number of clownfish associates and overall host anemone toxicity ranking. The curve represents a second order polynomial fit of r^2^ = 0.74. Anemone species within the same Genus represented by same shape symbol but with different colours (*Heteractis* spp. = circles; *Stichylodactyla* spp. = squares).

The relationship observed between number of anemonefish associates and overall host anemone toxicity ranking was found to be significant (r^2^ = 0.74; p = 0.018) based on a second order polynomial model fit (Figure 4). Host anemones with the lowest and highest overall toxicity ranking had the fewest anemonefish associates (Fig. 4) while species with a relatively moderate overall toxicity ranking had the greatest number of anemonefish associates.

## Discussion

Crude venom from nine anemone species that act as hosts for anemonefish showed significant differences in haemolytic and acute toxicity. Variation in relative neurotoxicity was also observed among anemone species. The neurotoxic effects on shore crabs displayed a range of effective paralytic doses with a 15-fold difference between the most potent (*E. quadricolor*, *H. aurora*, *S. haddoni* and *C. adhaesivum*) and least potent (*H. malu* and *M. doreensis*) anemones. Similarly, the dose range for *Artemia* toxicity was 100-fold and for haemolysis was 1000-fold which is consistent with the range of toxicities reported by Senčič and Maček [4] for a group of non-host anemone species. Our findings also compare well with a study by Mebs [3] on purified cytolytic toxins, showing that *H. magnifica* had a significantly greater haemolytic effect than *E. quadricolor* with a similar magnitude of difference between the EC_50_ concentrations. Our study differed from Mebs [3] who did not identify haemolytic activity for *H. crispa* venom. This difference may be due to a loss or reduction in toxin concentration during the purification process used by Mebs [3].

Although some dose response curves were classically sigmoidal, allowing a clearly defined 50% effective concentration to be determined, in other cases a non-sigmoidal character was observed. This may be a result of the crude venom containing a variety of active compounds of differing potency and/or mechanism of action [34] which has been shown for many anemone species elsewhere (see [1]). This may cause some deviations between true potency and the potency we have measured. However, the scale of these deviations appears to be small relative to the much larger variations between the venoms from different anemone species. Another factor that may have resulted in the underestimation of actual toxicity is the use of total protein of the crude venom as a relative measure of toxins. The crude venom would have contained non-venom proteins and thus the LD_50_ and EC_50_ venom concentrations reported may be higher than would result if the assays were conducted with the purified venoms. However, we believe the comparative potencies using crude venoms reported in this study are indicative of the toxicity of venoms delivered in nature.

Generally there was strong concordance between the rank toxicity of venoms in the three test approaches, suggesting that those with the highest haemolytic potential were also those that were more toxic to *Artemia*. There was somewhat less concordance between these two indicators and neurotoxicity although this was insufficient to reduce the statistical significance of the estimates. We may speculate that different mechanisms of toxic action may explain these findings and that further work is necessary to examine this possibility.

Toxicity ranking did not correspond to anemone phylogeny (see [35]). For example, most of the *Heteractis* species were found to be relatively low in haemolytic toxicity yet one congener, *H. magnifica,* had the second highest toxicity. Similarly, *S. gigantea* had a much lower haemolytic effect than *S. haddoni.* One study which may help to explain this lack of phylogenetic correspondence purified 2 cytolysins, Sticholysin I (St-I) and Sticholysin II (St-II) from the anemone *Stichodactyla helianthus*, and showed that despite a high degree of amino acid sequence homology (98%) the cytolysins differed significantly in their haemolytic activity [36]. Therefore, even small changes in the amino acid sequence can result in significant modifications of toxicity among related species.

The curved distribution characterising the relationship between overall anemone toxicity and number of anemonefish associates demonstrates that anemones with comparatively low or high toxicity, have fewer fish associates and anemone species with moderate toxicity have the highest number of fish symbionts. In fact, it appears that the most highly sought after anemones have a toxicity that falls within the middle of the toxicity range, suggesting that moderate toxicity may be optimal for anemonefish survival and reproduction.

High predation pressure in the tropical marine environment exerts strong selective pressure on sessile or slow-moving organisms to evolve defense mechanisms [37], [38]. Tropical anemones possess three defense strategies which may not be mutually exclusive; chemical defense, behavioural defense, and a symbiotic partner that provides protection. *C. adhaesivum* and *H. malu*, the anemone species which have a single anemonefish associate, are commonly found without anemonefish in the wild [39], [40]. This observation requires explanation as these anemone species cannot be solely relying on anemonefish for protection against grazers. When disturbed the least toxic anemone, *H. malu*, has the ability to completely withdraw beneath the sand ([39]; pers. ob.) and can remain hidden for at least 2 days. Conversely, *C. adhaesivum*, the most toxic anemone, does not exhibit this escape response when disturbed (pers. ob.). In this case the high toxicity level of *C. adhaesivum* may be sufficient as a defense strategy against grazers so that withdrawal under the sand is not required. It is likely that an upper toxicity threshold exists for anemonefish species to establish tolerance to venom without being harmed. As *C. adhaesivum* has only a single anemonefish symbiont living within it, might indicate that it is close to the upper limit. Lubbock [9] has suggested that *A. clarkii* (the only anemonefish known to associate with this most toxic anemone) has a thicker mucus layer than other anemonefish species, which may be an adaptation for this purpose.

Low anemone toxicity levels are also unlikely to be optimal for anemonefish survival. Interestingly, only juvenile anemonefish are known to associate with *H. malu,* the least toxic anemone in our sample. Similarly, *H. aurora*, also on the lower than average toxicity level only has juvenile anemonefish associates [22], [23], [15]. Juvenile anemonefish are far less likely to hold valuable resources when competition exists for anemones [18], therefore are likely excluded from high quality anemones and pushed into lower quality (and lower toxicity) anemone species. Similarly, Fautin [17], [18] suggests that *E. quadricolor* is the most desirable anemone host because it has the highest number of symbionts. Our results support this claim and suggest that *E. quadricolor* possesses optimal toxicity for anemoenfish survival which is further supported by the large number of highly competitive anemonefish species that use it, as well as the highly specialized anemonefish species that use it exclusively in the wild. The factors influencing survival are likely complex, but such a strong preference for *E. quadricolor* by anemonefish signifies the potential fitness benefits that higher quality anemones must afford their symbionts, and as Fautin [18] has shown competition for these valuable resources is high.

Models of parasite/host associations [41], [42] have shown that optimal hosts should increase the reproductive success of the symbiont and that host selection should reflect this. In the symbiotic relationship between anemonefish and anemones, this is not only true for reproductive success, but also overall fitness levels. According to Buston and Garcia [27] fishes within Amphiprioninae are extremely long-lived with life spans estimated to be six times greater than for marine tropical fish of similar size. If anemones are providing anemonefish protection then preference for high quality anemones should be expected to be under a high degree of selective pressure as seen above. Anemones are also required for protection of anemonefish eggs, which are laid beneath the anemone tentacles [28], and anemone toxins are used to deter egg predators such as wrasses [15].

An optimal toxicity range may be biologically significant for the establishment of anemonefish and anemone associations. Considering that anemonefish are not innately protected from anemone venom but have to acquire protection through a process of acclimation ([8]; pers. ob.), anemones that are highly toxic may be above a threshold for fish to acquire tolerance and are not used by anemonefish as hosts. Several authors have proposed that anemonefish mucus is involved in allowing anemonefish to survive within the toxic anemone environment (see review by Mebs [14]). Lubbock’s [9] finding that *A. clarkii* has a mucus layer three to four times thicker than other anemonefish species, may indicate a further adaptation to this more highly toxic environment. However, if toxicity is too low, anemonefish will not be able to obtain the benefits from the association. Similarly, these concerns relate to anemonefish oviposition sites, which require a toxicity balance between providing sufficient protection for developing embryos whilst not being harmful to the hatching larvae and may indicate that the two anemone species that only host juvenile anemone fish may not be suitable for embryo development.

Geographic distribution may explain some aspects of the pattern of fish and anemone association, in that areas where there are more fish species anemones will host more of them, but the analysis for fish was not consistent with this [43] suggesting distribution alone cannot account for the pattern that exists, although it may play a role.

This study has found that significant toxicity differences exist among venoms of nine sea anemone species that host anemonefish. Furthermore, it highlights a potential role of host anemone venom toxicity in the establishment and maintenance of symbiotic relationships and as a factor influencing anemonefish anemone selection behavior. Further comparative toxicological studies of host and non-host anemone species would allow a clearer delineation of optimal host toxicity and identification whether other preference criteria play a role in anemone use by anemonefish. Further studies addressing anemone choice will greatly contribute to our knowledge of host-symbiont co-evolution and increase our understanding of host utilization by anemonefish and other obligate anemone symbionts. Studies looking at interspecies variation in anemone toxicity and the toxicity of bleached or diseased anemones will be of particular interest with respect to anthropogenic change. If host anemone species diminish due to over collection or environmental pressures as discussed by Jones *et al*. [44], a fundamental question for the survival of anemonefish is whether they can utilize other anemone species as hosts. Anemone toxicity may be the limiting factor in anemonefish niche expansion but as shown in at least one anemonefish species, *A. clarkii*, utilization of anemone species with moderately higher toxicity may be an adaptation that will allow this unique symbiotic relationship to continue existing in the wild.
